# The association between tobacco control policy implementation and country-level socioeconomic factors in 31 European countries

**DOI:** 10.1038/s41598-021-88194-8

**Published:** 2021-04-26

**Authors:** Ariadna Feliu, Filippos T. Filippidis, Luk Joossens, Beladenta Amalia, Olena Tigova, Cristina Martínez, Esteve Fernández

**Affiliations:** 1 Tobacco Control Unit, WHO Collaborating Centre for Tobacco Control, Institut Català d’Oncologia (ICO), Av. Gran Via de L’Hospitalet 199-203, 08908 L’Hospitalet de Llobregat, Barcelona, Catalonia Spain; 2 School of Medicine and Health Sciences, Campus de Bellvitge, Universitat de Barcelona, L’Hospitalet de Llobregat, Barcelona, Catalonia Spain; 3 Tobacco Control Research Group, Epidemiology and Public Health Research Programme (EPIBELL), Institut d’Investigació Biomèdica de Bellvitge (IDIBELL), L’Hospitalet de Llobregat, Barcelona, Catalonia Spain; 4 Consortium for Biomedical Research in Respirarory Diseases (CIBERES), Madrid, Spain; 5 Department of Primary Care and Public Health, School of Public Health, Imperial College London, London, UK; 6 Tobacco Control Expert, Leuven, Belgium; 7 Philip R. Lee Institute for Health Policy Studies, University of California San Francisco, San Francisco, CA 94118 USA

**Keywords:** Risk factors, Public health, Epidemiology

## Abstract

European countries have made significant progress in implementing tobacco control policies to reduce tobacco use; however, whether socioeconomic status (SES) of a country may influence the implementation of such policies is unknown. The aim of this study is to assess the association between country-level SES and the implementation level of tobacco control policies in 31 European countries. An ecological study using data from Eurostat, Human Development Reports on several SES indicators and the Tobacco Control Scale (TCS) of 2016 was conducted to measure country-level tobacco control policies. We analysed the relationship between SES indicators and the TCS by means of scatter-plots and Spearman’s rank correlation coefficients (r_sp_) and multivariable linear regression analysis. In Europe, no statistically significant association was found between SES factors and the level of implementation of tobacco control policies. Only public spending on tobacco control was associated with all SES factors, except for Gini Index (an income inequality index). The strongest associations of TCS scores for this policy domain were found with the Human Development Index (r_sp_ = 0.586; *p* < 0.001) and the Gross Domestic Product *per capita* (in Euros) (r_sp_ = 0.562; *p* = 0.001). The adjusted linear regression model showed an association of tobacco control policy implementation with countries’ geographical location (Western Europe, β = − 15.7; *p* = 0.009, compared to Northern Europe). In conclusion, no association was found between SES factors and the level of implementation of tobacco control policies in 31 European countries; policymakers should be aware that tobacco control policies could be successfully implemented despite socioeconomic constraints, especially when these policies are of low cost and cost-effective (i.e., smoke-free bans and taxation).

## Introduction

Effective tobacco control policies are proven to denormalise smoking, resulting in a decrease of smoking prevalence^1^ and, consequently, in a reduction of tobacco-attributable morbidity and mortality^2^. Most European countries have increasingly implemented stringent tobacco control policies to reduce tobacco use and its negative consequences on health. However, tobacco consumption remains the largest avoidable health hazard^3^.

Tobacco control progress in Europe has been accelerated mainly by the enforcement of the WHO Framework Convention on Tobacco Control^4^ and, in the European Union (EU), by the Tobacco Products Directive, which entered into force in May 2014 and was transposed into national legislation by May 2016^5^. However, large differences still exist in the implementation and enforcement levels of tobacco control policies across Europe^6,7^.

Evidence on which factors influence governments to enact tobacco control policies is needed to understand why differences between European countries still exist. Multiple factors are potentially slowing the process of implementing policies that could reduce tobacco consumption at a country-level, including poor political commitment, tobacco industry interference or smuggling^8^. Previous studies have explored political factors such as corruption, political ideology, or governmental structure as drivers of tobacco control progress. Their results suggest that despite the modest influence that political factors have on tobacco control policy development; a strong and transparent governance are key to ensure that effective tobacco control policies are implemented^9,10^.

Income and education are associated with individual behaviours including smoking^11,12^, but also with perceptions and knowledge about smoking and tobacco control^13^. Therefore, it may be reasonable to suspect that such socioeconomic factors might play a role in developing and implementing policies also at the country-level. In this respect, we hypothesized that European countries with a higher socioeconomic status (SES) have a higher implementation level of tobacco control policies according to the six cost-effective measures assessed by means of the Tobacco Control Scale (TCS)^14^. Our aim was to assess the association between country-level SES and comprehensive tobacco control policy implementation in European countries.

## Methods

We conducted an ecological study with the country as the unit of analysis. We used data on tobacco control policies, measured by the TCS developed by Joossens and Raw^14^, for 31 out of the 35 European countries ranked in the TCS from 2016^15^, including 27 EU MS as well as four non-EU MS (Iceland, Norway, Turkey, and the United Kingdom). Serbia, Switzerland, the Russian Federation and Ukraine were excluded due to systematic missing data in the databases consulted.

Data on SES indicators in 2016 were obtained from two sources: the Eurostat^16^ and the Human Development Reports ^17^. The Eurostat is the statistical office of the EU aimed to provide high quality statistics at European-level using data from statistical systems of the countries. The Human Development Reports, which are commissioned by the United Nations (UN) Development Programme, obtains data from international data agencies. Both sources, however, harmonise national data using a consistent methodology to allow comparability across countries^16,18^.

## Variables

### Tobacco control policies

TCS scores were used to measure the implementation of tobacco control policies at a country-level in 2016. The scale is based on six cost-effective measures proposed by the World Bank that include dimensions such as: price (30 points), smoke-free laws (22 points), public spending on tobacco control (15 points), including mass communication campaigns, tobacco control projects, educational programs, and support for non-governmental organizations; tobacco advertising, sponsorship and promotion (TAPS) bans (13 points), health warnings (10 points) and treatment (10 points). The score given to each policy dimension is weighted by its reported effectiveness according to scientific evidence on tobacco control^19^. This score increases with the strength of tobacco control policies up to a possible maximum score of 100 points, indicating the highest level of implementation of all tobacco control measures considered, as each policy domain is rated individually^14^.

### Socioeconomic indicators

Countries’ SES can be defined by their income, wealth and poverty status, population educational level; and economic activity and working conditions^20^. Hence, we selected indicators that assess these SES factors and inequalities in 2016, including: human development index, wealth, people at risk of poverty, unemployment, long-term unemployment, educational level, income inequality, severe material deprivation, and gender inequalities (see Table 1).

**Table 1:** Summary of socioeconomic status (SES) indicators (description and data source).

Variable	Description	Data source
Human Development Index (HDI)	Summary measure of key dimensions of human development: a long and healthy life, being knowledgeable and a decent standard of living that is calculated through the geometric mean of normalised indices for each of the three dimensions	The United Nations Development Program Human Reports of 2016
Gross Domestic Product (GDP) per capita (in Euros)	GDP reflects the total value of all goods and services produced less the value of goods and services used for intermediate consumption in their production	Eurostat for the year 2016
Gini Index	Gini Index or coefficient is based on the comparison of cumulative proportions of the population against cumulative proportions of the income they receive, and it ranges between 0 in the case of perfect equality and 1 in the case of perfect inequality	Eurostat for the year 2016
People at risk of poverty	The percentage of people living in a household with an equivalised disposable income below the risk-of-poverty threshold, which is set at 60% of the national median equivalised disposable income (after social transfers)	Eurostat for the year 2016
Material Deprivation Index (MDI)	MDI expresses the inability to afford some items considered by most people to be desirable or even necessary to lead an adequate life. Severe material deprivation rate in percentage is defined as the enforced inability to pay for at least four of the deprivation items	Eurostat for the year 2016
Unemployment rate	The number of people of 15 to 74 years of age (16 to 74 years in Spain, Italy and the United Kingdom) that are not employed as a percentage of the active population	Eurostat for the year 2016
Long-term unemployment rate	Computed as the share of unemployed persons for 12 months or more in the total number of unemployed in the labour market	Eurostat for the year 2016
Gender Inequalities Index (GII)	GII measures gender inequality in three important aspects of human development: reproductive health, empowerment, and economic status	The United Nations Development Program Human Reports of 2016
Education Index	The Education Index is calculated using the mean years of schooling and expected years of schooling	The United Nations Development Program Human Reports of 2016

### Tobacco consumption

Prevalence of current adult smokers (over 15 years-old) in 2014 (data for the most recent year before 2016) was obtained from Eurostat^21^.

The male-to-female smoking prevalence ratio was calculated and included as an independent variable to proxy the stage of the tobacco epidemic^22^. Gallus et al*.* suggested that countries with a higher ratio within sexes are supposed to be in previous stages of the epidemic than those with a ratio closer to 1^23^.

### European regions

European countries were grouped in regions (Eastern, Northern, Southern and Western Europe) according to the geographic regions for Europe of the UN^24^.

### Statistical analysis

First, we calculated age- and sex-standardised smoking prevalence of current adult smokers for each country, by means of direct method of standardisation using the standard European population (year 2013). Then, we calculated the ratio between the age- and sex-standardised smoking prevalence in men and women in each country to obtain the male-to-female smoking prevalence ratio. We conducted a descriptive analysis calculating the mean, the standard deviation (SD) and the interquartile range (percentile 25 and 75) for all the variables of the study.

Second, we analysed the association between TCS scores (total and by its policy domains) (as dependent variable) and each of the SES indicators of European countries in 2016 (as independent variables) by means of scatter-plots and Spearman rank-correlation coefficients (r_sp_) and the corresponding 95% confidence intervals (CI).

Finally, we conducted a multivariable linear regression analysis to examine the association between TCS total scores and the SES indicators in 2016 in Europe. For model selection, we conducted a univariate linear regression for each SES indicators. Any variable having a significant univariate test at some arbitrary level (p-value cut-off point of 0.25) is selected as a candidate for the multivariate linear regression analysis following^25^. We used Akaike information criterion (AIC) to determine optimal specification of the linear regression and select the parameterized model with a higher efficiency. We performed statistical validation tests for all the models, which showed that the multivariable linear regression residuals that we fitted were appropriate with respect to the assumptions of linearity, normality of percentage point change and homoscedasticity; however, we observed that the variable Human Development Index (HDI) and Education Index and the Unemployment and Long-term Unemployment rates showed collinearity, according to the Variance Inflation Factor (VIF). Thus, we excluded the HDI from the model as it is a composite measure that includes components of the Gini and Education Indexes. In addition, we also excluded Unemployment rates as it is the denominator used to measure long-term unemployment as this indicator is measured as a percentage of unemployed. Analyses were performed with Stata 13.0 and SPSS 20.

### Ethical approval

Ethics approval for conducting this study based on secondary data from freely available databases was obtained from Hospital Universitari de Bellvitge [PR247/18].

## Results

Mean values (and SD) for dependent and independent variables are summarised in total and by European regions in Table 2. In Europe in 2016, the mean score of the level of tobacco control policy implementation according to TCS total score was 50.48 (10.58 SD), the top three ranking countries are the United Kingdom (TCS score of 81 out of 100 points), Ireland (70 points) and Iceland (69 points)^15^. Moreover, mean age- and sex-standardized smoking prevalence in Europe was 24.7% (4.66 SD) and the mean male-to-female smoking prevalence ratio was 1.57 (0.56 SD), both being higher in those countries with a TCS total score < 50 points.

**Table 2:** Summary of the age- and sex-standardized smoking prevalence, the male-to-female smoking prevalence ratio, and the socioeconomic (SES) indicators of 31 European countries (total and according to their TCS total score) in 2016.

Variables	Total	TCS < 50 points (n = 16)	TCS ≥ 50 points (n = 15)
	Mean (SD)	Mean (SD)	Mean (SD)
Smoking prevalence (%)	24.66 (4.66)	26.29 (0.01)	22.91 (0.01)
Male-to-female smoking prevalence ratio	1.57 (0.56)	1.64 (0.14)	1.49 (0.15)
Human Development Index	0.88 (0.04)	0.88 (0.01)	0.89 (0.01)
GDP per capita (thousand €)	29.54 (19.73)	26.60 (53.00)	32.69 (47.10)
Gini Index	30.27 (4.49)	30.47 (1.09)	30.05 (1.23)
People at risk of poverty (%)	23.82 (8.04)	24.03 (1.83)	23.59 (2.32)
Material Deprivation Index (%)	18.16 (12.48)	19.8 (3.04)	16.41 (3.35)
Unemployment (%)	8.15 (3.96)	9.04 (1.19)	7.19 (0.70)
Long-term unemployment (%)	3.80 (3.21)	4.51 (0.94)	3.05 (0.64)
Education Index	0.85 (0.06)	85.14 (1.27)	85.16 (1.77)
Gender Inequality Index	0.12 (0.08)	0.11 (0.01)	0.13 (0.03)

SD: Standard deviation; GDP: Gross Domestic Product.

TCS total scores were categorized into two categories using the median value (P50 = 49) as cutting point.

**Figure 1:**
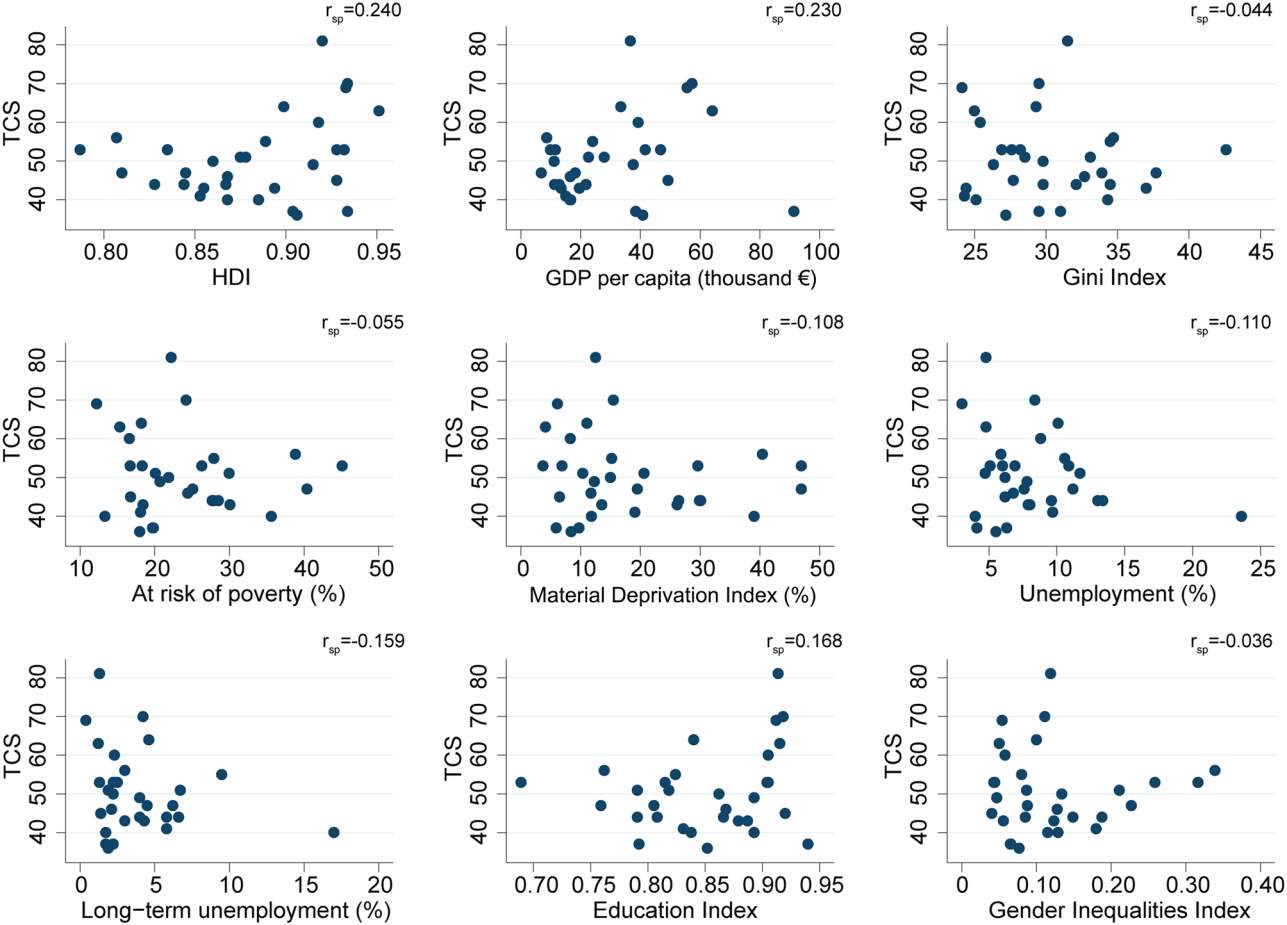
Correlation between Tobacco Control Scale (TCS) total scores and socioeconomic status (SES) indicators in 2016 in 31 European countries. *GDP: Gross Domestic Product, HDI: Human Development Index, MDI: Material Deprivation Index. r_sp_: Spearman-rank correlation coefficient.

At the ecological level, no correlations with a *p* < 0.05 were found between SES indicators and TCS total score (Fig. 1). However, as shown in Table 3, the countries’ scores on public spending on tobacco control showed a moderate to strong association with most of the SES indicators, except for the Gini Index and both variables related to unemployment. The strongest associations were found with HDI (r_sp_ = 0.586 (0.286 to 0.781); *p* < 0.001) and GDP per capita (in Euros) (r_sp_ = 0.562 (0.252 to 0.767); *p* = 0.001).

**Table 3:** Spearman’s rank-correlation coefficients (r_sp_) and 95% confidence intervals (CI) between socioeconomic status (SES) indicators and Tobacco Control Scale (TCS) scores in 2016 (overall and by components) in 31 European countries.

	SES Factors (n = 31)								
**TCS**	HDI	GDP	Gini Index	At-risk of poverty	MDI	% Unemployment	% Long-term unemployment	Education Index	Gender Inequalities Index
Total	0.240 (− 0.125; 0.548)	0.230 (− 0.163; 0.520)	− 0.044 (− 0.393; 0.315)	− 0.055 (− 0.401; 0.306)	− 0.108 (− 0.445; 0.256)	− 0.110 (− 0.447; 0.254)	− 0.159 (− 0.486; 0.207)	0.168 (− 0.198; 0.493)	− 0.036 (− 0.386; 0.322)
	*0.194*	*0.274*	*0.813*	*0.771*	*0.563*	*0.555*	*0.392*	*0.366*	*0.846*
Price	0.048 (− 0.312; 0.396)	0.001 (− 0.354; 0.355)	0.081 (− 0.282; 0.423)	0.130 (− 0.235; 0.463)	0.190 (− 0.176; 0.510)	− 0.003 (− 0.357; 0.352)	− 0.033 (− 0.383; 0.325)	− 0.007 (− 0.361; 0.348)	0.205 (− 0.161; 0.521)
	*0.798*	*0.997*	*0.667*	*0.485*	*0.306*	*0.988*	*0.858*	*0.970*	*0.269*
Smoke-free laws	0.251 (− 0.114; 0.556)	0.251 (− 0.114; 0.556)	0.019 (− 0.338; 0.371)	0.013 (− 0.343; 0.366)	− 0.129 (− 0.462; 0.236)	− 0.114 (− 0.450; 0.250)	− 0.156 (− 0.484; 0.210)	0.148 (− 0.218; 0.477)	− 0.065 (− 0.410; 0.296)
	*0.174*	*0.174*	*0.921*	*0.943*	*0.489*	*0.541*	*0.401*	*0.427*	*0.729*
Public spending	**0.586 (0.286; 0.781)**	**0.562 (0.252; 0.767)**	− 0.292 (− 0.590; 0.076)	− 0.376 (− 0.648; − 0.018)	− 0.455 (− 0.700; − 0.113)	− 0.135 (− 0.472; 0.237)	− 0.227 (− 0.543; 0.145)	**0.467 (0.129; 0.708)**	− **0.476 (**− **0.714; **− **0.140)**
	< *0.001*	*0.001*	*0.117*	*0.041*	*0.011*	*0.478*	*0.227*	*0.009*	*0.008*
TAPS bans	0.194 (− 0.172; 0.513)	0.205 (− 0.161; 0.521)	− 0.240 (− 0,547; 0,125)	− 0.214 (− 0.528; 0.152)	− 0.192 (− 0.512; 0.174)	− 0.057 (− 0.403; 0.304)	− 0.125 (− 0.459; 0.240)	0.154 (− 0.212; 0.482)	− 0.023 (− 0.375; 0.334)
	*0.295*	*0.269*	*0.194*	*0.248*	*0.300*	*0.763*	*0.502*	*0.409*	*0.901*
Health warnings	− 0.021 (− 0.373; 0.336)	− 0.136 (− 0.468; 0.230)	0.144 (− 0.221; 0.474)	0.049 (− 0.311; 0.396)	0.050 (− 0.309; 0.398)	− 0.129 (− 0.462; 0.236)	− 0.122 (− 0.457; 0.242)	0.153 (− 0.213; 0.481)	0.256 (− 0.108; 0.560)
	*0.910*	*0.466*	*0.439*	*0.796*	*0.788*	*0.489*	*0.512*	*0.411*	*0.164*
Treatment	0.230 (− 0.136; 0.540)	0.286 (− − 0.076; 0.581)	0.057 (− 0.303; 0.403)	− 0.008 (− 0.362; 0.347)	− 0.133 (− 0.465; 0.232)	0.105 (− 0.259; 0.443)	0.032 (− 0.326; 0.382)	0.059 (− 0.302; 0.405)	− 0.220 (− 0.532; 0,146)
	*0.214*	*0.119*	*0.760*	*0.964*	*0.476*	*0.573*	*0.863*	*0.752*	*0.235*

Note: Bold, *p* value < 0.01. TAPS: Tobacco, advertising, promotion, and sponsorship; HDI: Human Development Index; GDP: Gross-domestic Product per capita (in Euros); MDI: Material Deprivation Index.

As shown in Table 4, crude linear regression models showed that the TCS total score in 2016 was 9.8 (*p* = 0.039) and 11.4 (*p* = 0.034) points lower in Southern and Western Europe, respectively; compared to Northern Europe. The multivariable linear regression model showed that only 31.4% (*p* = 0.076; Table 4) of the TCS total score(s) in 2016 was explained by SES indicators after adjusting for male-to-female smoking prevalence ratio and countries’ geographical region. Our adjusted results found that countries with higher Education Index (ß = − 0.40; *p* = 0.382) and male-to-female smoking prevalence ratio (ß = − 10.31; *p* = 0.069) had on average lower TCS total score(s), although neither relationships were statistically significant. Western Europe countries scored 15.69 points less on average in the total TCS than Northern countries (*p* = 0.009) (Table 4).

**Table 4:** Crude and adjusted multivariate linear regression models examining the association between SES indicators and the overall Tobacco Control Scale (TCS) score of 2016 in 31 European countries.

	Crude		Adjusted	
	β (SE)	*p* value	β (SE)	*p* value
**HDI** (100 points)	0.732 (0.437)	0.104		
**GDP** per capita (1000 €)	0.127 (0.097)	0.199		
**Unemployment**	− 0.618 (0.483)	0.211		
**Long-term unemployment**	− 0.779 (0.595)	0.201		
**Education Index** (100 points)	0.396 (0.324)	0.232	− 0.402 (0.452)	0.382
**Male-to-female ratio**				
≤ 1.33	REF		REF	
> 1.33	− 6.037 (3.702)	0.114	− 10.311 (5.416)	0.069
**Region**				
Northern Europe^1^	REF		REF	
Eastern Europe^2^	− 9.567 (5.108)	0.072	− 5.249 (6.349)	0.416
Southern Europe^3^	− 9.844 (4.545)	**0.039**	− 8.921 (6.137)	0.158
Western Europe^4^	− 11.4 (5.108)	**0.034**	− 15.690 (5.538)	**0.009**
R-squared			0.314	0.076

The crude linear regression model shows the results of a simple linear regression model of the dependent variable and the independent variable. HDI and Unemployment were excluded due to collinearity with the Education Index and Long-term unemployment, respectively. The adjusted multivariable regression model assessed the effect of SES indicators (Education Index) adjusted for male-to-female ratio and geographical region. We performed the adjusted analysis with the model with the optimal AIC values and fulfilled all regression validation tests. Male-to-female ratio is presented as a binary variable after categorizing countries using the median value (1.33) as cutting point as it fitted better the linear regression model.

HDI: Human Development Index; GDP: Gross Domestic Product.

1: Denmark, Estonia, Finland, Iceland, Ireland, Latvia, Lithuania, Norway, Sweden, United Kingdom; 2: Bulgaria, Czech Rep., Hungary, Poland, Romania, Slovakia; 3: Croatia, Cyprus, Greece, Italy, Malta, Portugal, Slovenia, Spain, Turkey; 4: Austria, Belgium, France, Germany, Luxembourg, and the Netherlands.

SE: Standard error.

## Discussion

### Main findings

Overall, no associations were found between the selected direct indicators of countries’ SES factors and their implementation level of tobacco control policies according to TCS scores, except for public spending on tobacco control. Differences in tobacco control efforts between European countries may partly be explained by their male-to-female ratio (used as a proxy of the stage of the epidemic at which countries are) and a geographical component^23^.

### Interpretation of the results

Our results do not show an association between the level of the wealth of a country and the implementation level of tobacco control policies as no statistically significant associations were found regarding SES indicators. Hence, although smoking has proven to be a burden for the poor, our results could not confirm that in Europe SES inequalities at national-level are associated with an implementation of tobacco control policies. However, although our findings do not show any relationship between SES and tobacco control policy implementation, the width of reported 95% CI for Spearman correlations, which include values of 0.5 or − 0.5, was large enough to suggest that our results might underestimate a relationship between them. However, by using an ecological design we were not able to either evince it or explain it. Therefore, further policy research on tobacco control is needed to determine whether high SES are a prerequisite for comprehensive tobacco control policies or not, which we know have the potential to reduce smoking inequalities, if they continue for a long term, covering and reaching all socioeconomic subgroups^26^. Accordingly, previous studies have identified several low-income countries that have also succeeded to implement effective tobacco control policies^13^. Further research is needed to characterize the cultural, social, and ideological factors that drive progress on tobacco control that is key to advance in the field. It is probable that in order to unveil some of these drivers of policy implementation according to SES in Europe we will need to employ multimethod combining qualitative and quantitative research^27^.

There was a moderate to strong association between countries’ public spending on tobacco control and almost all SES indicators, except for Gini Index and those related to unemployment. Our results suggest that the wealthier the country, the higher the amount spent by governments in tobacco control per capita. Accordingly, European countries with higher SES seem to have invested more on mass media campaigns, tobacco control projects, educational programs, and support for non-governmental organizations. A plausible explanation could lay on the fact that high-quality anti-tobacco campaigns are expensive to produce and broadcast^26^ since successful use of mass media requires sustained campaigns with broad population reach, which includes keeping campaigns “on the air” most months of the year^27^.

The economic recession, which affected European countries for several years after 2008, further complicates our analysis. Many of the tobacco control policies which were in place in 2016 were actually implemented before 2008. Countries, such as Spain, Italy or Ireland were still recovering from the economic crisis^28^ in 2016. Thus, the SES indicators used in the analysis may not entirely capture the socioeconomic conditions at the time when some of these policies were first implemented, with varying differences across countries. For example, Spain where the GDP per capita in Purchasing Power Standards –that is expressed in relation to EU28 average set to equal 100 –has decreased from 101 in 2008 when the first tobacco control law entered into force to 91 in 2016^29^.

## Limitations and strengths

This is an ecological study, and consequently, any causal relationship between national SES and the implementation of tobacco control policies in European countries is difficult to establish. However, we are not trying to infer the relationship at the individual-level but simply assessing an ecological effect.

The small sample size (n = 31) of the study could be another potential limitation as it reduces the statistical power of the analysis; albeit our study includes all the countries ranked in the TCS report of 2016 (n = 35), except those with systematic missing data for SES indicators. These exclusions could have entailed an underestimation of the association in the subset of countries included in our study, because all four countries excluded have a TCS score under 50 points^15^; and two are upper middle-income countries (Serbia and the Russian Federation) and one is a lower middle-income country (Ukraine). Moreover, our sample may not be representative of the WHO European Region, which consists of 53 countries, especially of Eastern Europe. Another limitation is the lack of a clear indicator to establish the tobacco epidemic stage^22^ across the different countries. In addition, as the attributable mortality rates were not available for all countries, we could not make an approximation of these stages; however, and with the available data in our hands, we calculated the male-to-female smoking prevalence ratio as a proxy of the epidemic stage, and then we were in the position to control our analysis for this potential confounder.

The use of the TCS as a measure of tobacco control policy implementation in European countries also has some limitations since it is based on policy enactment and not their enforcement^15^, except for smoke-free policies. Additionally, the dimension public spending on tobacco control has some missing data. However, this scale has been useful in several evaluations of tobacco control and different health outcomes performance^30^.

Another limitation to be noted is the possible bidirectional association between implementing tobacco control policies and SES. It is not clear that countries with strong tobacco control policies may have decreased social, economic and health inequalities at the population level. Price increases and targeted population-level cessation support continue to be the only interventions where there is consistent evidence of a greater effect among low-SES smokers^31^. Nevertheless, due to the ecological nature of our study we are not able to establish this association that should be assessed through longitudinal studies.

Our study is the first to assess the association between tobacco control policies implementation and countries’ SES to better understand the large differences that still exist in the implementation and enforcement levels of tobacco control policies across Europe.

## Conclusions

Tobacco control policy implementation in Europe—according to our results –has not been found to be associated with SES indicators at a country-level. However, the SES effects on tobacco control policy implementation could not be absolutely discarded by using an ecological design. Further longitudinal policy research is needed to understand how SES factors affect policymakers’ decisions on whether to implement or not population-based tobacco control measures. Less costly policies including smoke-free places or tobacco products taxation have shown to be effective to reduce smoking prevalence.
